# F241. DEVELOPMENT AND VALIDATION OF THE SOCIAL FUNCTIONING ASSESSMENT SCALE (SFAS) FOR PEOPLE WITH SCHIZOPHRENIA IN TURKEY

**DOI:** 10.1093/schbul/sby017.772

**Published:** 2018-04-01

**Authors:** Mustafa Yildiz, Fatma Kiras, Aysel İncedere, Duygu Esen, Mehmet Buğrahan Gürcan, Betül Abut, Kübra İpçi, Güzide Örüm, Ümit Tural, Güliz Özgen

**Affiliations:** 1 Kocaeli Universitesi; 2 Bakirkoy Ruh Sagligi ve Hastaliklari Hastanesi

## Abstract

**Background:**

Social functioning is generally defined as having profound and qualitative interpersonal relations, and meeting the expectations and defined roles in society. Determining the level of the social functioning is important especially for people with schizophrenia that proceed with disabilities. Main dimensions of the social functioning are 1) Self-care, 2) Independent living, 3) Interpersonal relationships (family, friends, neighbors, etc.), 4) Leisure time and recreation, and 5) Occupational activities like school or job. The purpose of this study was to develop a culturally-sensitive, user-friendly scale that could assess the social functioning of the people with schizophrenia.

**Methods:**

After examining the studies assessed social functioning in people with mental illnesses, an original 50-item scale was formed. Habits of 425 people living in the community was examined with this form so as identifying the prevalence and frequency of behavior patterns related with social functioning in Turkish community. Regarding the findings of that study, 28-item scale was formed that assess the social functioning of the patients. New form was given to 25 patients, and items which was difficult to comprehend were reevaluated and the scale was finalized as Social Functioning Assessment Scale. One hundred and thirty outpatients with schizophrenia or schizoaffective disorder were given a sociodemographic form, Social Functioning Assessment Scale (SFAS), Clinical Global Impression-Severity (CGI-S), and Global Assessment of Functioning (GAF). At the same time, Social Functioning Scale (SFS) and SFAS was given to the relatives of the patients who live together. For reliability analyses; internal consistency coefficient, item-total correlation, and split-half reliability was assessed. For validity analyses; explanatory factor analysis, and convergent validity were examined via Spearman correlation.

**Results:**

The data from 104 patients with schizophrenia and 26 with schizoaffective disorder whose 75% were males, 69% were single, mean age was 37, the level of education was 10 years was examined. The average onset of the illness was 23 years, and the duration of illness was 14 years. Cronbach’s alpha coefficient for SFAS total score was .83, and for factors were between .69 and .77. Split-half reliability coefficient of SFAS was .73. There was a satisfactory correlation between SFAS filled by patients and by relatives (r=.60, p<0.001). For factor analysis, Kaiser-Meyer-Olkin value was .78, and Barlett test was significant (p<0.001). In explanatory factor analysis, SFAS was found to be compose of three factors (self-care, interpersonal relationships and recreation, independent living) and that they can explain 45% of the total variance. Nine items were omitted because of having lower factor value than .40. Self-care factor had 7-item, interpersonal relationships and recreation factor had 7 items and independent living factor had 4 items. Occupational life could not get in any of factors; however, since it was very important for social functioning, it was added to the scale as fourth factor.

SFAS total score was correlated with PANSS negative subscale (r=-.35, p<0,001), PANSS-total (r=-.29, p<0,001), CGI-S (r=-.33, p<0,001), GAF (r=.28, p<0,001) and SFS total score (r=.52, p<0,001).

**Discussion:**

Regarding the findings of the study, SFAS was considered a culturally-sensitive, easy-to-use, and valid instrument that objectively assesses the social functioning of the patients with schizophrenia in Turkey.

